# Electrophoretic Deposition of Chitosan/45S5 Bioactive Glass Composite Coatings Doped with Zn and Sr

**DOI:** 10.3389/fbioe.2015.00159

**Published:** 2015-10-19

**Authors:** Marta Miola, Enrica Verné, Francesca Elisa Ciraldo, Luis Cordero-Arias, Aldo R. Boccaccini

**Affiliations:** ^1^Department of Applied Science and Technology, Politecnico di Torino, Turin, Italy; ^2^Department of Materials Science and Engineering, Institute of Biomaterials, University of Erlangen-Nuremberg, Erlangen, Germany

**Keywords:** electrophoretic deposition, bioactive glasses, composite coatings, therapeutic ions, chitosan

## Abstract

In this research work, the original 45S5 bioactive glass was modified by introducing zinc and/or strontium oxide (6 mol%) in place of calcium oxide. Sr was added for its ability to stimulate bone formation and Zn for its role in bone metabolism, antibacterial properties, and anti-inflammatory effect. The glasses were produced by means of melting and quenching process. SEM and XRD analyses evidenced that Zr and Sr introduction did not modify the glass structure and morphology while compositional analysis (EDS) demonstrated the effective incorporation of these elements in the glass network. Bioactivity test in simulated body fluid (SBF) up to 1 month evidenced a reduced bioactivity kinetics for Zn-doped glasses. Doped glasses were combined with chitosan to produce organic/inorganic composite coatings on stainless steel AISI 316L by electrophoretic deposition (EPD). Two EPD processes were considered for coating development, namely direct current EPD (DC-EPD) and alternating current EPD (AC-EPD). The stability of the suspension was analyzed and the deposition parameters were optimized. Tape and bending tests demonstrated a good coating-substrate adhesion for coatings containing 45S5-Sr and 45S5-ZnSr glasses, whereas the adhesion to the substrate decreased by using 45S5-Zn glass. FTIR analyses demonstrated the composite nature of coatings and SEM observations indicated that glass particles were well integrated in the polymeric matrix, the coatings were fairly homogeneous and free of cracks; moreover, the AC-EPD technique provided better results than DC-EPD in terms of coating quality. SEM, XRD analyses, and Raman spectroscopy, performed after bioactivity test in SBF solution, confirmed the bioactive behavior of 45S5-Sr-containing coating while coatings containing Zn exhibited no hydroxyapatite formation.

## Introduction

Metallic alloys (such as Ti alloys, stainless steel, or Co–Cr alloys) are the materials of choice for bone implants where good mechanical properties and load-carrying ability are required (Niinomi, 2002; Chen and Thouas, 2015). These materials generally are osteointegrated by morphological fixation, i.e., by bone ingrowth on their surface irregularities by press fitting into a bone defect or by cementing the implant with acrylic bone cement. Metallic alloys generally lack osteoinductive properties and, if the bone ingrowth is not effective, they could develop a fast formation of a non-adherent fibrous capsule at the interface which, in turn, can cause bone interfacial loosening and the failure of the implant. A better behavior can be achieved if a bioactive fixation is induced, i.e., a direct chemical bonding with the bone (Drnovšek et al., 2012). For this purpose, the modification of the implant surface with a bioactive coating (i.e., a coating with the ability of forming a biologically active hydroxyapatite layer on its surface) can lead to a significant improvement of its osteointegration. Bioactive glasses (BGs) and glass-ceramics can be successfully used with this purpose (Rahaman et al., 2012; Jones, 2013; Hench, 2015) due to their peculiar ability of inducing bone ingrowth. BGs have been studied and developed during the last 40 years for a vast number of medical applications, including powders, granules, 3D scaffolds, additive for injectable or putty-like bone substitutes, second phases in bone cements, as well as dense coatings on metallic and ceramic substrates (Gerhardt and Boccaccini, 2010; Jones, 2013). Moreover, BG compositions can be easily modified by introducing several elements with therapeutic effect (Mouriño et al., 2012); for example, Ag, Cu, and Ga have been added for their antimicrobial effect, Cu was used for its ability to stimulate angiogenesis and bone (Miola et al., 2014), and Mn for its role in the metabolism of muscle and bone (Rath et al., 2014). Among the investigated elements, also Zn and Sr have drawn the attention of the researchers for their role in bone metabolism and the Zn antibacterial effect (Balamurugan et al., 2007; Gentleman et al., 2010; Jaiswala et al., 2012; Balasubramanian et al., 2015).

Bioactive ceramic coatings can be applied on the bioinert implant surface by means of several methods, such as glazing, enameling, plasma praying, electrophoretic deposition (EPD), RF-magnetron sputtering, and pulsed laser deposition (Verné, 2012).

Electrophoretic deposition (Besra and Liu, 2007) has been gaining interest in different sectors for its versatility and its cost-effectiveness and EPD of biomaterials is being increasingly investigated (Boccaccini et al., 2010). In particular, the advantages of EPD are its applicability to a wide range of materials (ceramics, polymers, metals, and composites), the use of simple equipment, and the possibility to deposit homogenous thin or thick coatings on substrates with different shapes and dimensions in a matter of minutes. Moreover, EPD can be carried out using aqueous and non-aqueous solvents (Besra and Liu, 2007). Even if water-based EPD implies some problems due to the electrolysis phenomenon, it has been shown that using relatively high voltages and alternating current EPD (AC-EPD), it is possible to obtain high-quality coatings and films (Neirinck et al., 2009; Chávez-Valdez and Boccaccini, 2012).

Since the deposition of pure ceramics can lead to the formation of a brittle coating, they often are codeposited with polymers, especially natural polymers (Cordero-Arias et al., 2014). Among biopolymers, chitosan, a linear, semicrystalline polysaccharide, shows outstanding properties: biocompatibility, the ability to complexes various species such as metal ions, pH-dependant solubility, low cost, and antibacterial activity. Moreover, chitosan is a suitable film-forming polymer that does not require further heat treatment (Croisier and Jérôme, 2013). For these reasons, chitosan is often used in EPD process to realize coatings suitable for orthopedic applications and bone tissue engineering (Pishbin et al., 2011; Cordero-Arias et al., 2013).

In this article, 45S5-base BGs doped with Zn and Sr were combined with chitosan to produce organic/inorganic composite coatings on stainless steel AISI 316L by direct current (DC)-EPD and AC-EPD. The effects of the added ions on the glass and coatings bioactivity combined with the influence of the two EPD methods on coating properties were investigated.

## Materials and Methods

### Glasses Synthesis and Characterization

Zinc oxide (6 mol%), strontium oxide (6 mol%), and both Zn and Sr oxide (ZnO, 3 mol%; SrO, 3 mol%) were added to the original 45S5 BG composition (Hench, 2006) by replacing calcium oxide; the doped glasses were named 45S5-Zn, 45S5-Sr, and 45S5-ZnSr and their compositions are presented in Table 1.

**Table 1:** **Compositions of the 45S5-based bioactive glasses investigated**.

%wt	45S5	45S5-Sr	45S5-Zn	45S5-Sr-Zn
SiO_2_	45.00	43.01	43.92	43.46
Na_2_O	24.50	23.42	23.91	23.66
P_2_O_5_	6.00	5.73	5.86	5.79
CaO	24.50	18.19	18.58	18.38
SrO	0.00	9.65	0.00	4.87
ZnO	0.00	0.00	7.74	3.83

All the glasses were produced by means of melting and quenching process: the reactants were accurately mixed and subsequently put in a Pt crucible maintained at 1500°C for 30 min. The melt was then poured in water obtaining a frit, which was dried at room temperature overnight and then milled and sieved to reach a grain size <20 μm.

The influence of Zn and Sr on the glass structure was evaluated by X-ray diffraction (XRD – X’Pert Philips diffractometer), using the Bragg Brentano camera geometry and the Cu-Ka incident radiation; the obtained pattern was analyzed with X’Pert High Score software and the PCPDF data bank. All glasses were also analyzed from the morphological and compositional points of view by Field Emission Scanning Electron Microscopy (FESEM – SUPRATM 40, Zeiss) equipped with energy-dispersive spectroscopy (EDS).

In order to assess the effect of the introduced oxides on the mechanism of bioactivity, glass powders were immersed in SBF (Kokubo and Takadama, 2006) for different time periods (1, 3, 7, 14, 21, and 28 days). The test was performed in an orbital shaker (KS 4000i control, IKA^®^) at 37°C using a glass powder (mg)/SBF (ml) ratio of 1 and an agitation rate of 120 rpm (Magallanes-Perdomo et al., 2012). At the end of the incubation time, the powders were washed with water and acetone, filtered, dried during 3 h at 60°C, and analyzed by means of XRD and FESEM-EDS. For comparison, the bioactivity of the original 45S5 BG was evaluated for up to 3 days.

### Suspension Preparation and Stability Evaluation

Doped glass powders and chitosan were used to produce organic/inorganic composite coatings on AISI 316L stainless steel substrate (15 mm × 20 mm) by EPD, using both direct current (DC) and alternating current (AC). Suspensions containing 0.5 g/L chitosan (80 kDa, 85% deacetylation, Sigma) and 1.5 g/L glass powders were prepared in a solvent mixture of 1 vol% acetic acid (Sigma-Aldrich), 20 vol% pure water (Purelab Option R7BP, ELGA), and 79 vol% ethanol (VWR). These experimental parameters were chosen according to previously reported results (Grandfield and Zhitomirsky, 2008; Cordero-Arias et al., 2013). Before the EPD process, the suspensions were sonicated with a VWR USC 300 sonicator for 1 h and stirred during 5 min to avoid particle sedimentation. Suspension stability was evaluated by measuring the ζ-potential, using a Zetasizer nano ZS equipment (Malverb Instrument, UK).

### Coatings Deposition and Characterization

Different deposition parameters were investigated. For the DC-EPD process, the deposition time varied between 30 s and 2 min and the electric potential in a range of 20–80 V. Regarding the AC-EPD, the voltage varied in the range of 8–20 V and the deposition time between 1 and 2 min; moreover, a frequency range of 4–10 KHz and a duty cycle of 70–80% were considered. For both, DC- and AC-EPD procedures, the distance between the deposition and counter electrodes was kept constant at 10 mm. The choice of the optimal parameters was carried out on the basis of morphological analysis of the samples by means of the coating homogeneity in preliminary experiments. Coating microstructure was evaluated by SEM (Hitachi S4800) while FTIR (Nicolet 6700) analysis was carried out to determine the presence of chitosan and the different glass particles in the coating.

The mechanical characterization of the coatings was performed through the tape test (four samples for each composition), in accordance with ASTM D3359-09 standard, and a qualitative flexural bend test by manually flexing the sample up to an angle of 180° according to previous studies (two samples for each composition) (Chen et al., 2013; Cordero-Arias et al., 2013, 2014). The deposited mass was calculated by weighing the samples before and after the deposition process, both using AC- and DC-EPD methods. Chitosan/45S5-Sr coatings were selected as an example, and 10 measures were performed for each deposition by means of a precision balance (0.0001 g).

The surface roughness (Ra) of all coatings, synthesized both using AC-EPD and DC-EPD, was measured by means of a laser profilometer (UBM, ISC-2); the analysis was performed in triplicate. The coating wettability was estimated through static contact angle measurements using a DSA30 instrument (Kruess GmbH, Germany). Five measurements for each sample were carried out using distilled water.

Bioactivity test in SBF was performed according to Kokubo’s protocol (Kokubo and Takadama, 2006) to evaluate the influence of the doping elements on the final bioactivity of the coating. Coated samples were immersed in 40 ml of SBF solution maintained at 37°C for 1, 3, 7, 14, 21, and 28 days. Every 7 days, the SBF solution was refreshed. After the immersion period, samples were analyzed by means of SEM, XRD, and Raman spectroscopy (LabRAM HR800, Horiba Jobin Yvon) to evaluate the formation of hydroxyapatite (HAp) or its precursor on the samples surfaces.

## Results

### Glasses Synthesis and Characterization

**Figure 1:**
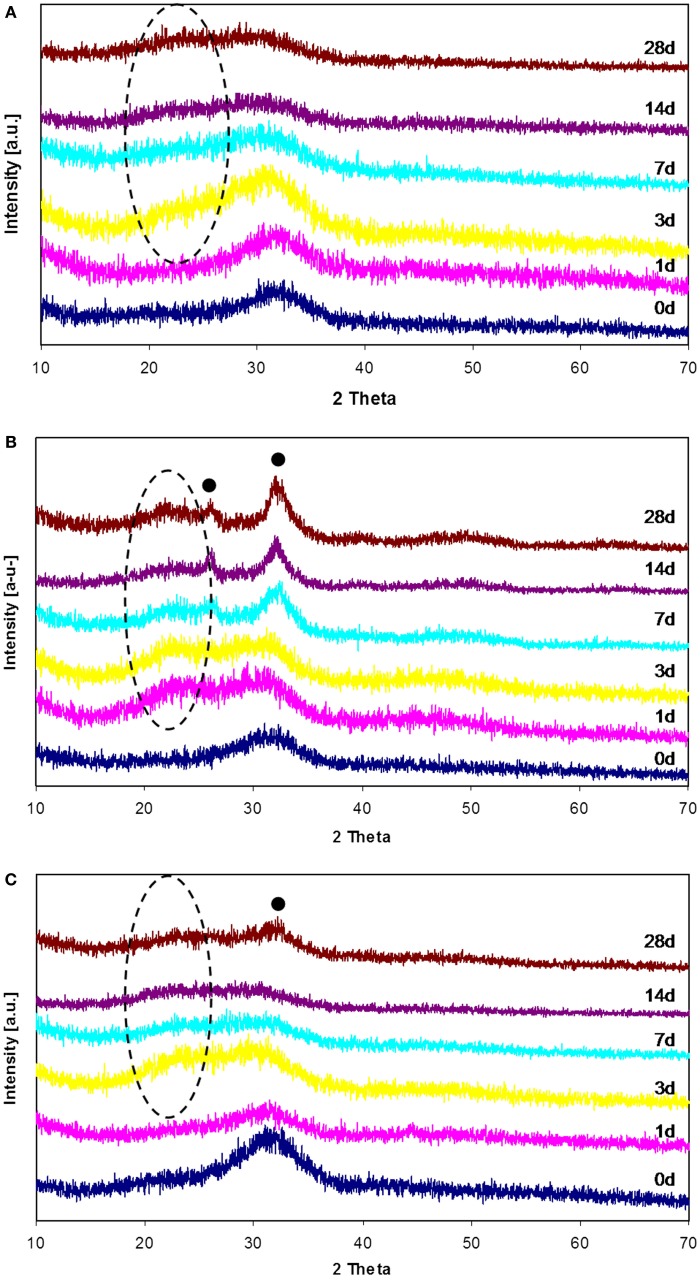
**XRD patterns of 45S5-Zn (A), 45S5-Sr (B), and 45S5-ZnSr (C) before and after SBF treatment up to 1 month**. (•) Hydroxyapatite, (–) silica gel.

The structural analyses of Zn- and/or Sr-doped glasses (Figure 1) demonstrated that all glass powders were amorphous; therefore, the introduction of Zn and Sr oxides did not induce nucleation of crystalline phases. Also from the morphological point of view, no significant differences were evidenced by FESEM analyses while the presence of doping elements was verified by means of EDS analysis (data not shown here).

Immersion tests in SBF solution revealed the influence of Sr and Zn on bioactivity of the glasses. In particular, Zn-containing glasses showed a delay in the nucleation of HAp, even if the silica gel formation and its enrichment in Ca and P were observed after few days of SBF immersion.

**Figure 2:**
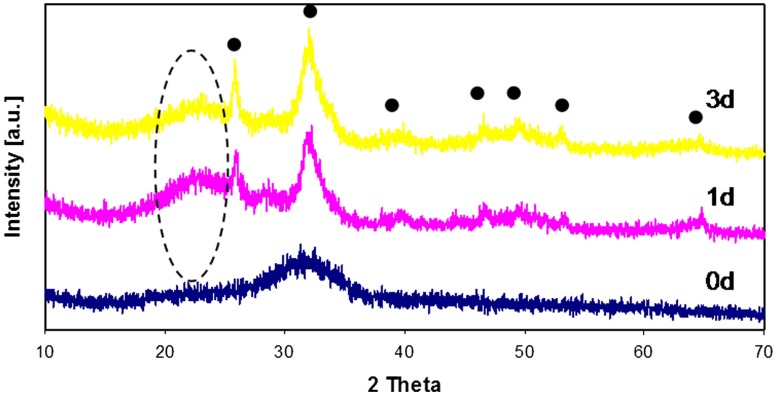
**XRD patterns of 45S5 (BG) before and after SBF treatment up to 3 days**. (•) Hydroxyapatite, (–) silica gel.

Figure 1 reports the XRD patterns of all glasses before and after SBF treatment up to 1 month; as it can be noticed, no crystallization peaks were observed for 45S5-Zn glasses (Figure 1A), but only the typical silica-gel halo at about 2 theta = 20°–25° after 3 days of immersion in SBF was noticed. XRD analyses of 45S5-Sr glasses (Figure 1B) showed always the presence of the silica-gel halo after 1 day of treatment and the appearance of HAp peaks after 7 days of immersion in SBF; then in this case only a slight delay, in comparison to the pristine glass (45S5 BG, Figure 2), in the HAp crystallization was observed. Glasses containing both Zn and Sr showed an intermediate behavior: silica gel was present after 3 days of SBF immersion, but the HAp peaks appeared only after 1 month of treatment.

**Figure 3:**
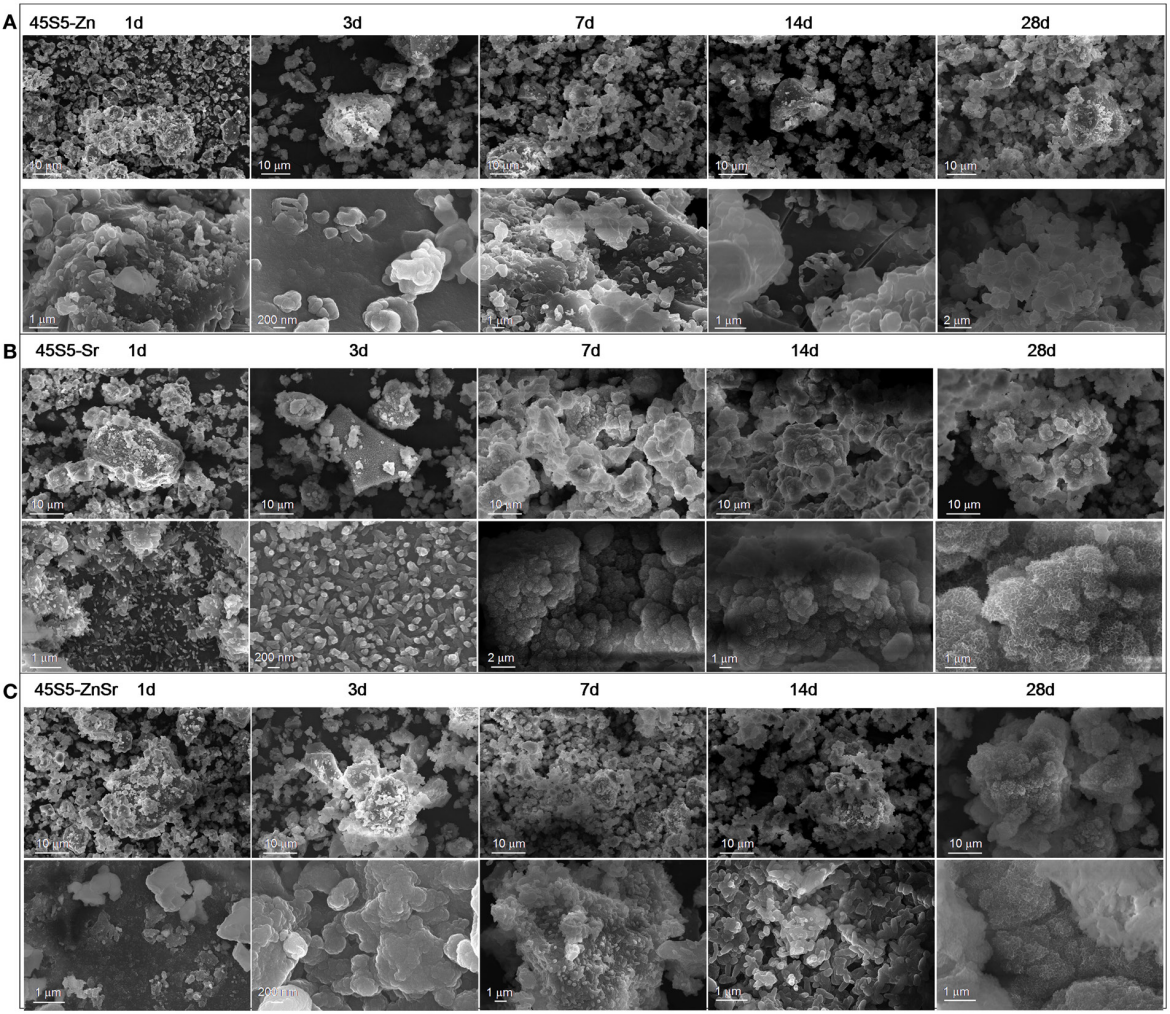
**SEM micrographs of 45S5-Zn (A), 45S5-Sr (B), and 45S5-ZnSr (C) after different times of immersion in SBF**.

**Figure 4:**
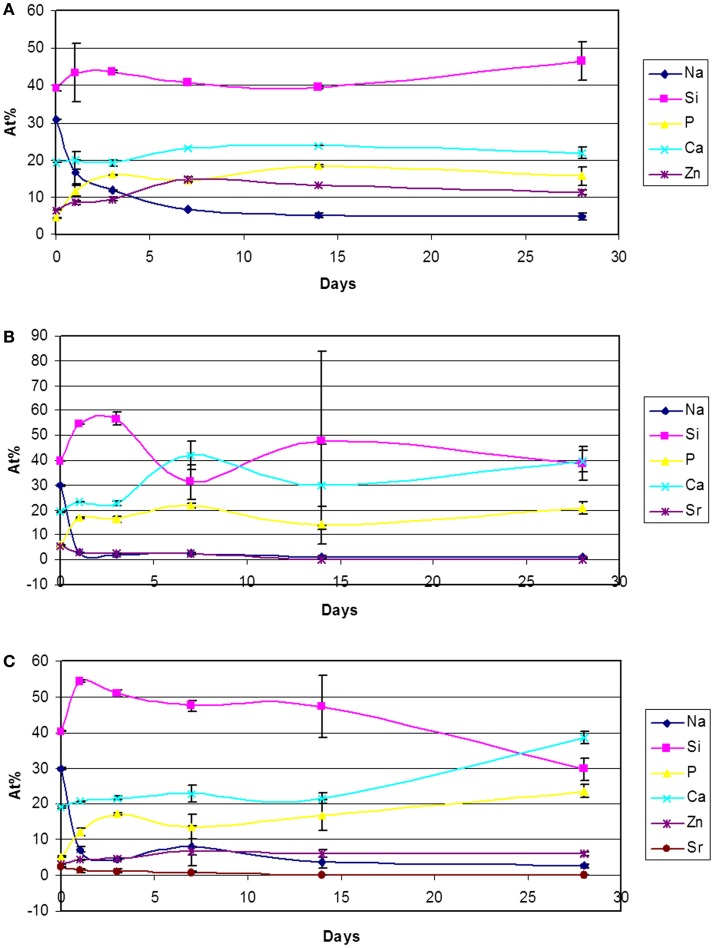
**Atomic percentages variation (EDS analyses of 500× area) of 45S5-Zn (A), 45S5-Sr (B), and 45S5-ZnSr (C) after immersion in SBF**.

The results obtained by means of XRD analyses were confirmed by FESEM observation and EDS analyses, reported in Figures 3 and 4 respectively. Regarding 45S5-Zn glass powders, no formation of crystals with the typical globular shape of HAp was evidenced up to 1 month, even if a reaction layer can be noticed on SEM images (Figure 3A) and an increase of Ca and most of all P was evidenced by EDS analysis (Figure 4A). A noticeable increase of Ca and P concentration together with the decrease of Na were also evidenced for 45S5-Sr glass (Figure 4B). SEM analyses revealed the presence of globular agglomerates with the typical morphology of *in vitro* self-grown HAp (Kaur et al., 2014) after 7 days of SBF treatment. Also in this case, 45S5-ZnSr presents an intermediate performance: EDS analysis showed a significant increase of Ca and P and a reduction of Na concentrations, which are intermediate between those of 45S5-Sr and 45S5-Zn. Moreover, globular precipitates were confirmed to form after 28 days of immersion by means of SEM observation.

### Suspension Stability

The ζ-potential results for BG suspension stability are presented in Table 2. For the Sr- and Zn-doped BGs/chitosan suspensions, the ζ-potential results show a similar value. All systems exhibit a relative high standard deviation (14 mV), and this result can be related to the large size of the glass particles decreasing the suspension stability. Nevertheless, a cathodic deposition is predicted for all the systems. It can be assumed that deposition is controlled by the chitosan molecules, which move the potential from a negative value for the BG to a positive value when chitosan is incorporated, this occurs by the esterification that the polymer chain has on the surface of the BG particles, as discussed in previous studies (Zhitomirsky et al., 2009; Pishbin et al., 2011; Chen et al., 2013; Cordero-Arias et al., 2013).

**Table 2:** **ζ-potential results for suspensions containing the different doped bioactive glasses in a mixture with chitosan**.

Suspension	ζ-Potential (mV)
45S5-Sr/Ch	+29 ± 14
45S5-Zn/Ch	+31 ± 15
45S5-ZnSr/Ch	+36 ± 14

The fresh prepared suspensions exhibited a pH value in the range of 3–4.5 for all suspensions. Under those conditions, the suspensions were used for EPD.

### Coating Synthesis and Characterization

**Figure 5:**
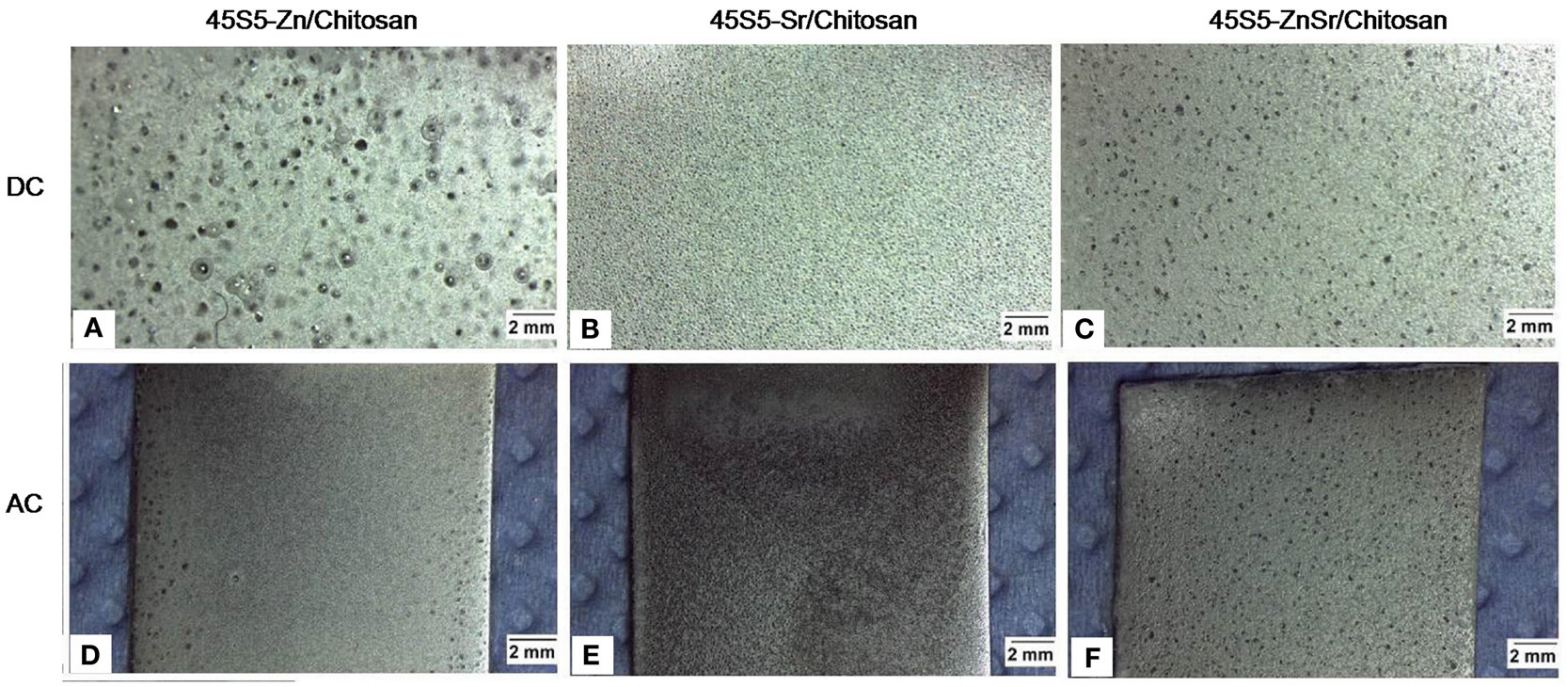
**Images of the electrophoretic coatings using both DC (A–C) and AC-EPD (D–F) for the different chitosan/BG systems investigated**.

Using the DC-EPD technique, homogeneous and well-attached coatings were obtained for the 45S5-Sr- and 45S5-ZnSr-based coatings using 75 V and 1 min of deposition potential and time, respectively (Figures 5B,C). In the case of the 45S5-Zn/Ch system, the coatings presented a higher porosity and microcracks, which are likely due to a stronger hydrolysis during the deposition (Figure 5A). When AC-EPD was used, homogeneous and well-attached coatings were obtained for the different glasses using 20 V of deposition potential, 2 min of deposition time, 10 kHz of frequency, and a duty charge of 80% (Figures 5D–F). Comparing the 45S5-Zn/Ch produced by DC-EPD and AC-EPD, better coatings were produced using the AC-EPD method since the voltage is periodically inverted, the supplied energy is not sufficient to cause water hydrolysis, and the coatings appeared more homogeneous. These results are in accordance with previously reported data (Neirinck et al., 2009; Chávez-Valdez and Boccaccini, 2012; Chen et al., 2013). The used voltage did not have influence on the deposition of 45S5-Sr powders, allowing a uniform deposition both in AC and DC processes. Finally, in 45S5-ZnSr-containing coating, a low amount of bubbles was detected, but there were no differences between AC and DC deposition.

**Figure 6:**
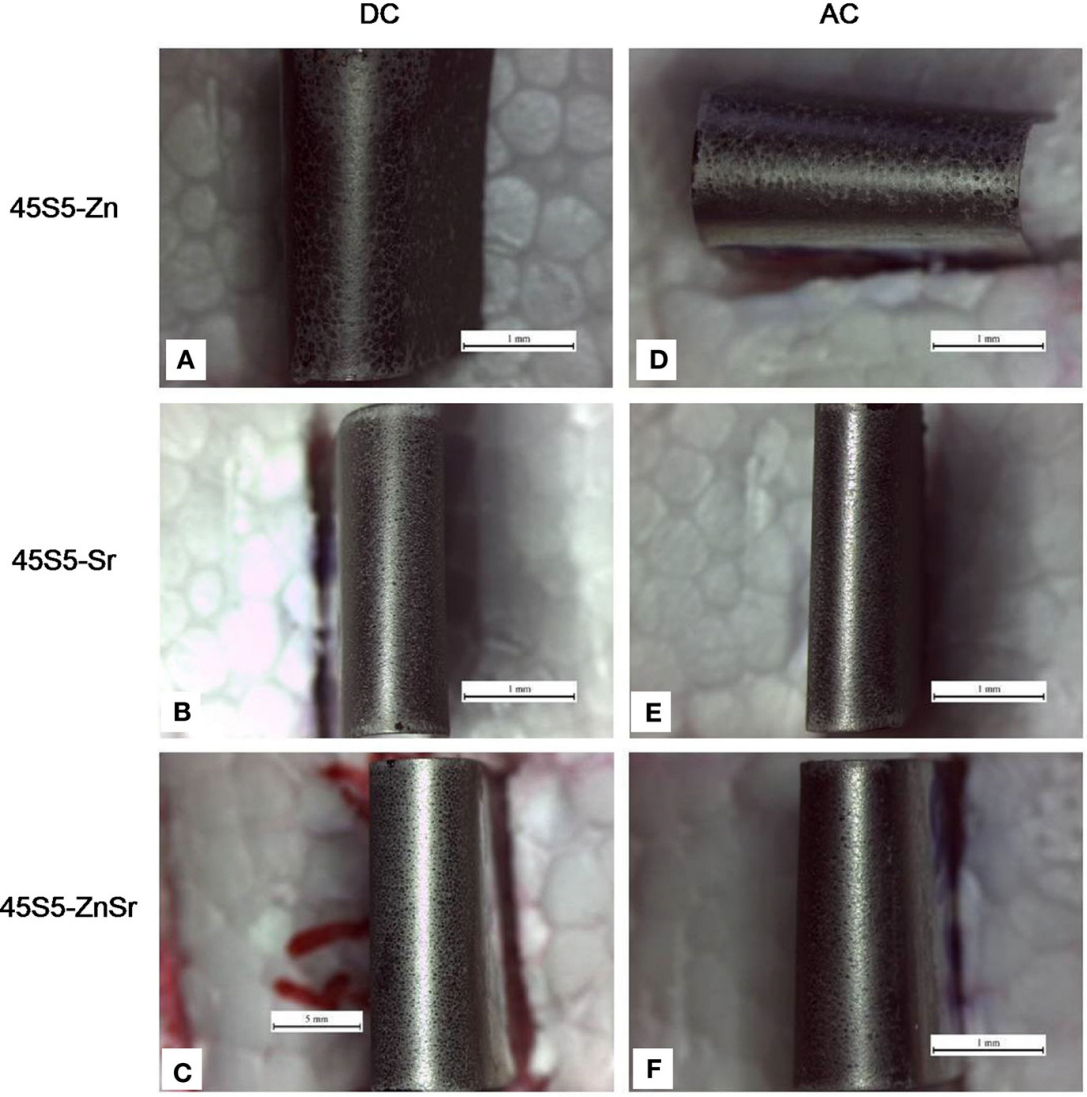
**Digital camera images of the surface of coatings after bending tests**. Coatings produced by means of DC-EPD **(A–C)** and AC-EPD **(D–F)**. Scale bar: 1 mm.

The qualitative bending test demonstrated an optimal adhesion of all coatings on the substrates, without the formation of cracks or detachments (Figure 6).

Concerning the measurement of deposited mass, AC-EPD led to a deposited mass of 1.3 ± 0.2 mg/cm^2^ while 0.8 ± 0.2 mg/cm^2^ was calculated for DC-EPD coatings.

**Figure 7:**
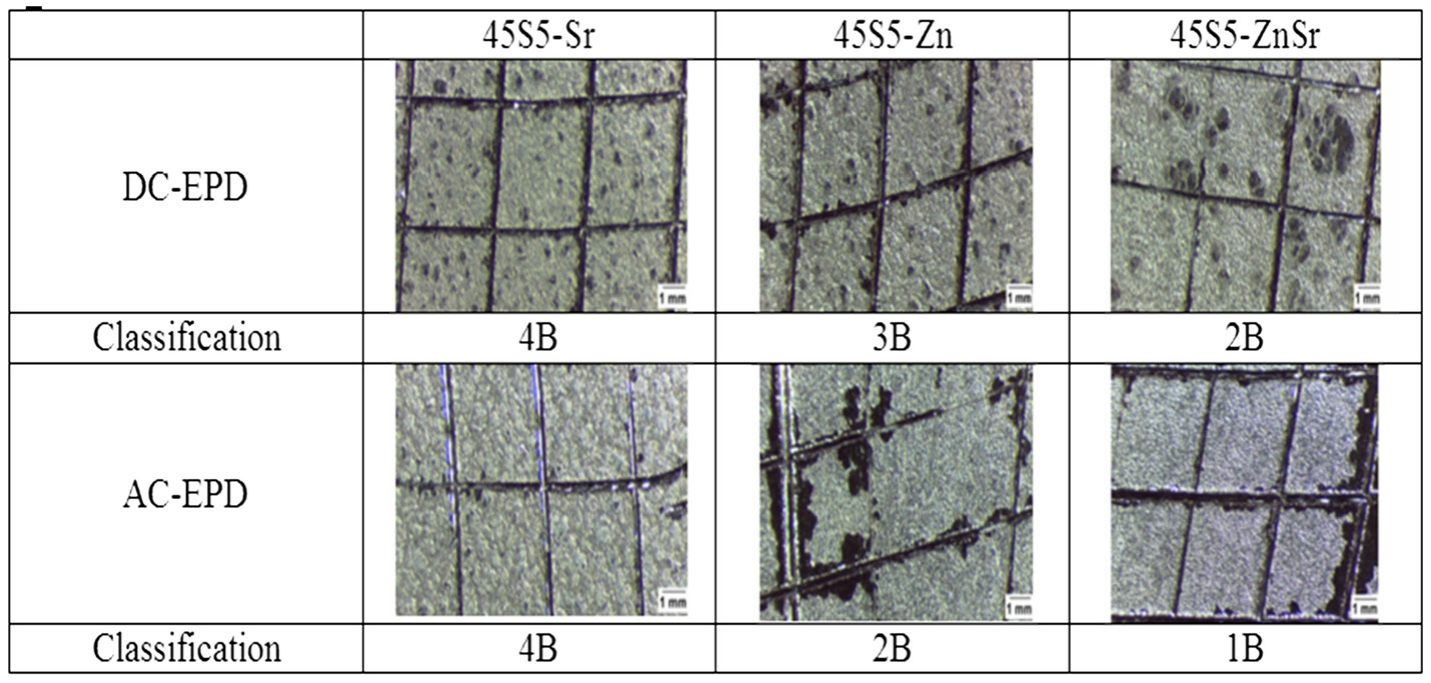
**Tape test results for the different chitosan/45S5-doped BG coatings on stainless steel AISI 316L substrate confirming different adhesion behavior of coatings depending on deposition mode (AC- or DC-EPD)**.

The tape test (Figure 7), performed in accordance with ASTM D3359-09, evidenced some differences among the various coatings; the 45S5-Zn coatings showed a damage classified as 2B for AC-EPD (65% of the coating removed) and 3B for DC-EPD (25% of the coating removed). 45S5-Sr coatings revealed a damage <5% for both deposition modes and were classified as 4B. Finally, 45S5-ZnSr coatings showed a significant damage both for AC-EPD (classified as 1B, more than 65% of the coating removed) and for DC-EPD (classified as 2B, 65% of the coating removed).

**Figure 8:**
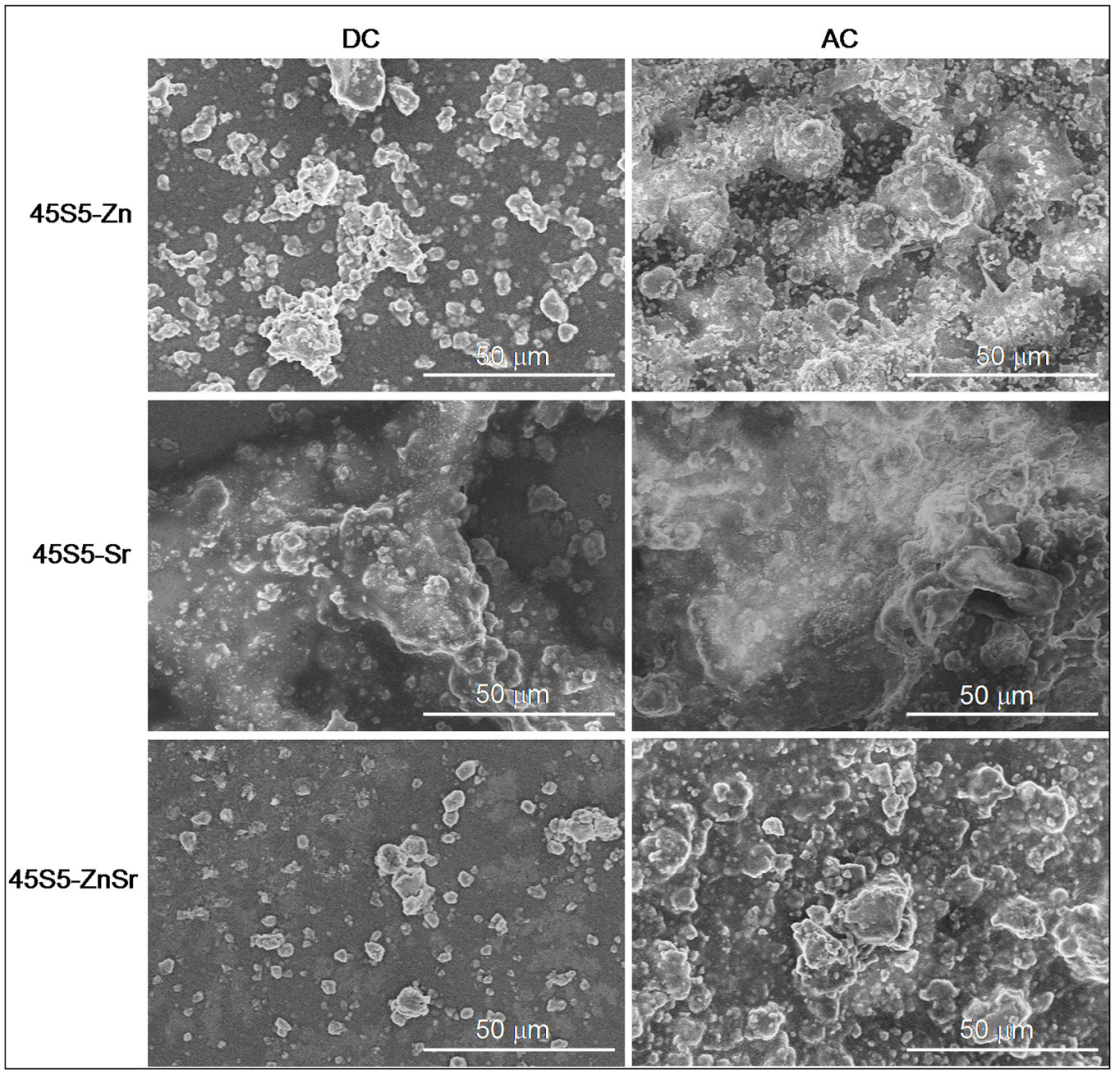
**SEM images of AC- and DC-EPD-obtained coatings showing glass particles in chitosan matrix**.

The morphological analysis of the coatings is reported in Figure 8. As it can be observed, the 45S5-Zn coatings exhibit poor homogeneity, and the glass particles formed clusters which are well embedded in the polymeric matrix. Regarding the deposition process, AC-EPD allowed depositing glass particles/clusters with greater dimensions than the DC-EPD process, covering more of the substrate surface. 45S5-Sr coatings appear relatively more homogenous and uniform with glass particles well integrated in the chitosan matrix. No substantial difference between DC- and AC-EPD is observed in terms of microstructure. Concerning the 45S5-ZnSr coatings, a more pronounced difference was noticed between DC and AC deposition; the coatings obtained with DC-EPD are clearly less consistent and homogenous in comparison to those synthesized with AC-EPD, similar results for DC- and AC-EPD for organic/inorganic coatings have been previously reported (Chen et al., 2013).

**Figure 9:**
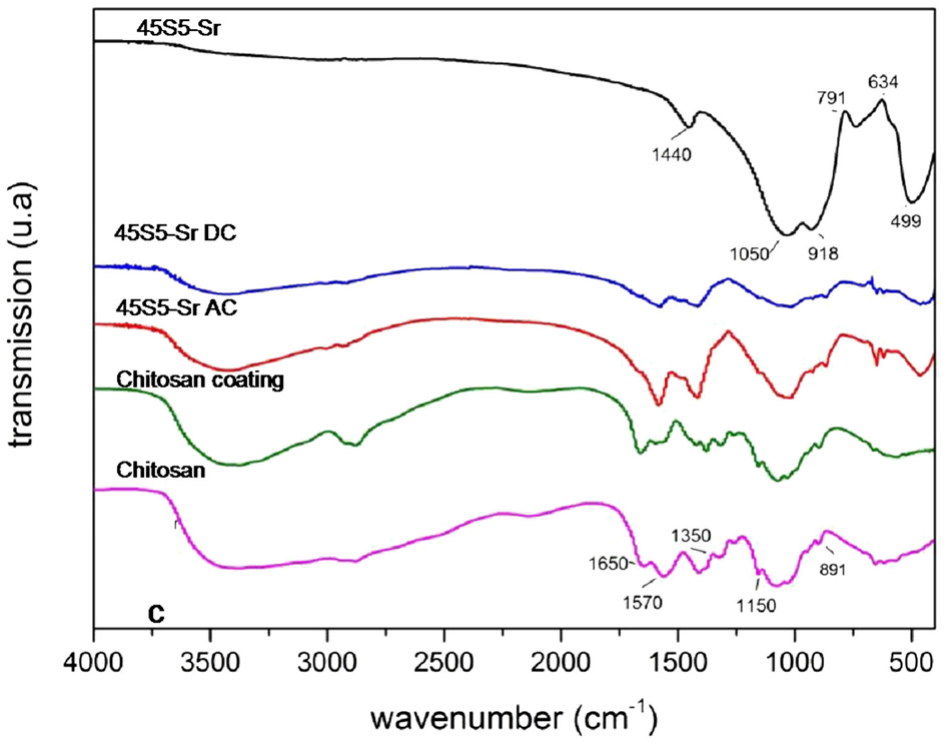
**FTIR spectra of chitosan/45S5-Sr**. The relevant peaks are explained in the text.

Figure 9 shows as example the FTIR spectrum of an Sr-containing coating. In order to verify the presence of chitosan, the FTIR spectrum of pure chitosan powder was obtained together with that of pure glass powder (45S5-Sr). Moreover, also the spectrum of a chitosan coating was obtained.

FTIR analysis evidenced the composite nature of all coatings. The chitosan characteristic peaks were present in all spectra, in particular the peaks around 891 cm^−1^ and 1150 cm^−1^, which correspond to the saccharide structure and are due to the vibrations of the glycosidic bonding (–C–O–C–). The stretching of the C=O group in the glucosamine unit appears at about 1650 cm^−1^ and corresponds to the amide ι band. The band at 1570 cm^−1^ corresponds to the amide II band, indicating the NH-bending vibrations in the amide group (Gebhardt et al., 2012). Finally, the peak at 1350 cm^−1^ is ascribable to the vibration of the C–H/N–H group. Instead, the characteristic bands of BG are located at about 916 cm^−1^ and 496 cm^−1^ and correspond to the vibration of Si–O–Si and P–O groups, respectively.

The roughness of the coatings was evaluated and the obtained values are reported in Table 3. As it can be observed, the roughness values are generally higher for coatings obtained by AC-EPD process. This behavior is probably due to the grater amount of material deposited.

**Table 3:** **Roughness (Ra) and contact angle values of the different produced coatings**.

	45S5-Zn AC	45S5-Zn DC	45S5-Sr AC	45S5-Sr DC	45S5-ZnSr AC	45S5-ZnSr DC
Ra (μm)	1.8 ± 0.2	1.6 ± 0.08	2.2 ± 0.4	1.2 ± 0.1	1.6 ± 0.04	1.7 ± 0.2
Contact angle	47° ± 5°	54° ± 9°	41° ± 6°	62° ± 12°	73° ± 7°	59° ± 8°

Table 3 reports also the results of wettability test; the values of the contact angle measurements varied between 35° and 80°. As reported in the literature (Chen et al., 2013), the presence of BG should lead to hydrophilic properties, and hydrophilic surfaces are promising for the *in vitro/in vivo* cells interaction.

**Figure 10:**
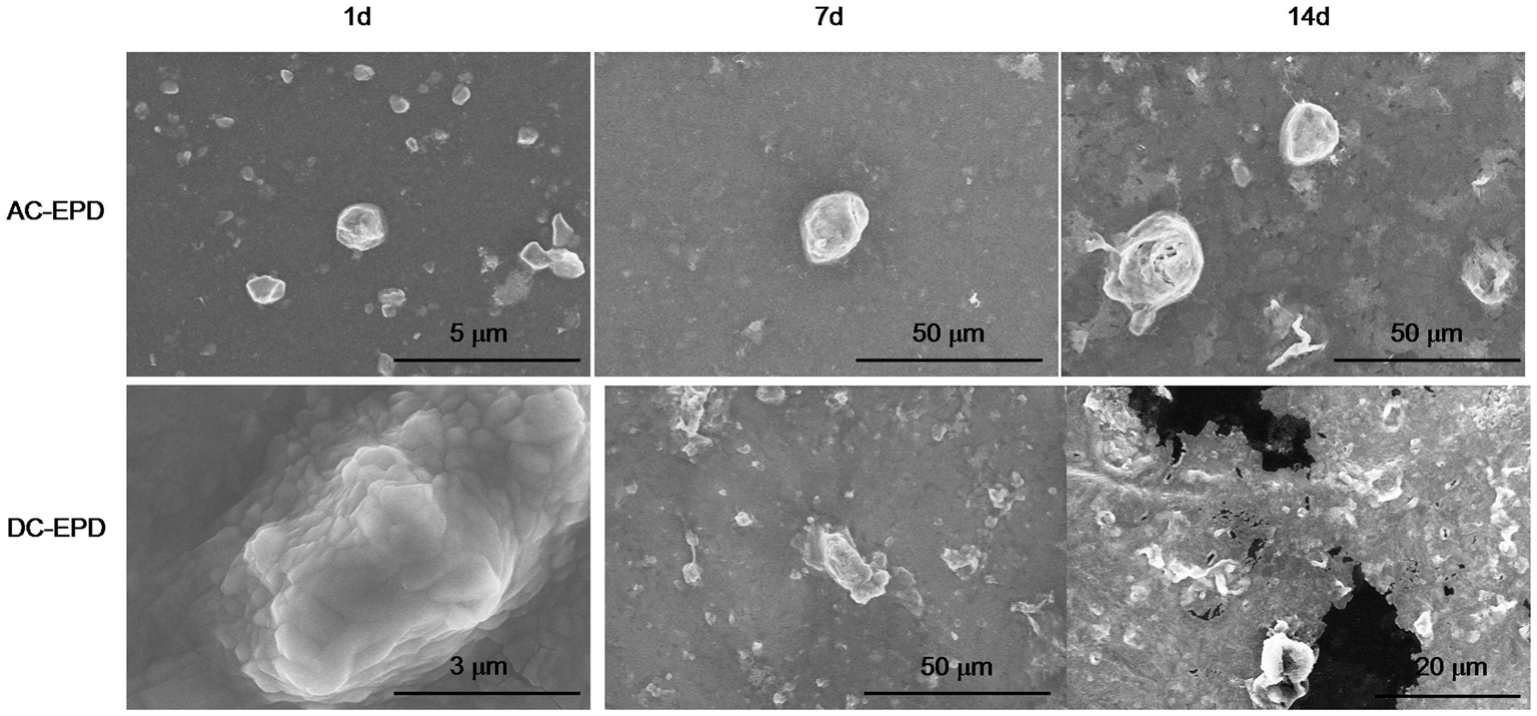
**SEM micrographs of 45S5-Zn-containing coatings after different times of immersion in SBF**.

Figure 10 shows the results obtained for 45S5-Zn-containing coating upon immersion in SBF. SEM images confirmed the results obtained on glass powders: the Zn presence causes a pronounced inhibition of the precipitation of HAp. No differences were evidenced between AC- and DC-EPD modes at any time of immersion in SBF.

**Figure 11:**
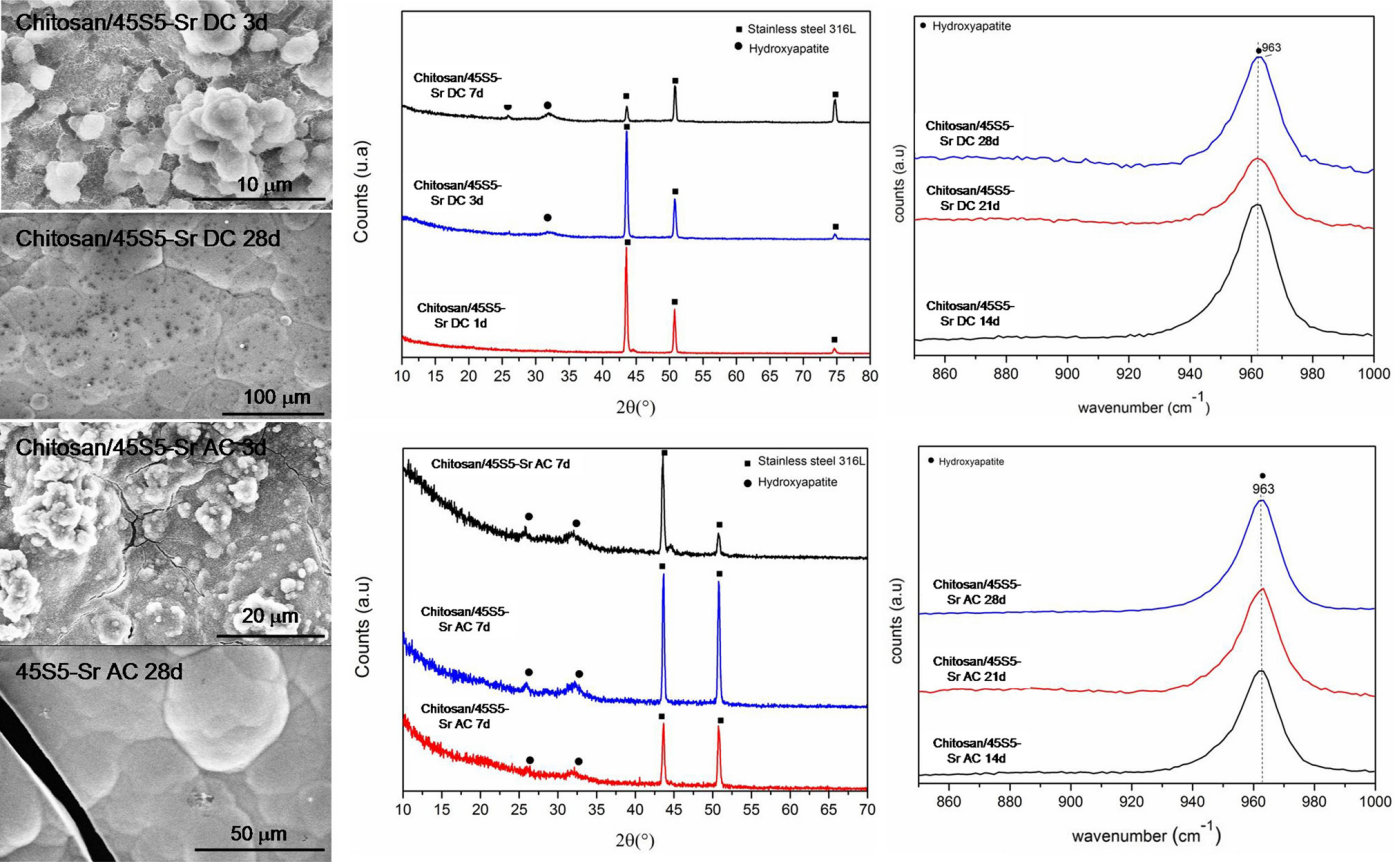
**SEM, XRD, and Raman analyses of 45S5-Sr up to 1 month of SBF immersion**.

On the contrary, Sr-containing coatings were able to induce the precipitation of HAp after 1 day for AC-EPD and 3 days for DC-EPD coatings. Figure 11 shows SEM micrographs, XRD, and Raman analyses of the samples: globular agglomerates similar to *in vitro* grown HAp are visible after few days of treatment; the HAp presence is confirmed by XRD up to 7 days and Raman analyses from 7 to 28 days (Koutsopoulos, 2002; Li et al., 2004).

**Figure 12:**
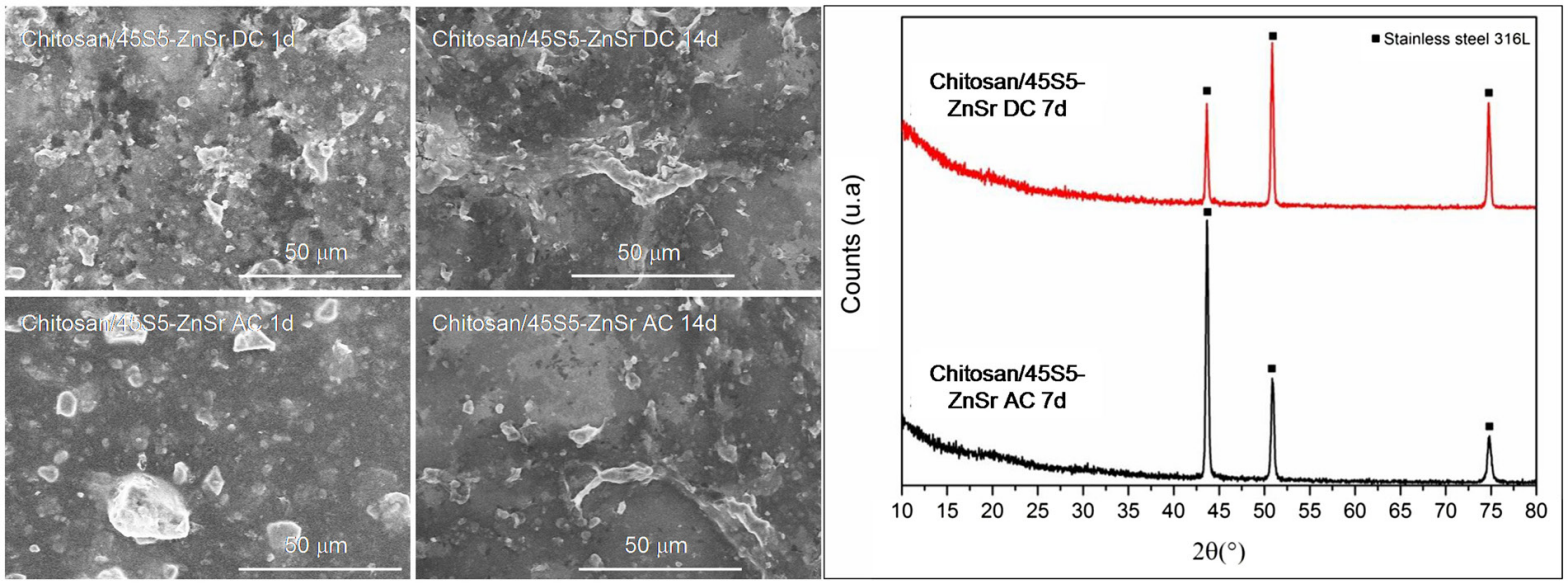
**SEM micrographs and XRD analyses of 45S5-ZnSr after immersion in SBF solution**.

Finally, a reduced bioactivity was also evidenced for 45S5-ZnSr-containing coatings, since up to 14 days no HAp precipitation was detected both with SEM and XRD analyses (Figure 12).

## Discussion

This research has considered the synthesis of Zn- and Sr-doped glasses and their use to develop chitosan/glass composite coatings by means of EPD. In particular, the influence of Zn, Sr, and their combination on the bioactivity process was investigated from both glass powders and coatings. Moreover, the effect of these ions in the EPD process was also evaluated.

Regarding the glass powders, both Zn and Sr introduction did not change the structure of the pristine glass (45S5), since no crystallization peaks were detected in XRD spectra. However, the incorporation of these elements, especially Zn, generated differences on the kinetics of bioactivity. In fact, Zn-doped glass (45S5-Zn) did not show the precipitation of crystalline HAp up to 1 month of immersion in SBF solution (Figure 1A). The influence of Zn on the bioactivity process and its role in glass structure have been extensively debated in literature, as reviewed recently (Balasubramanian et al., 2015). It has been reported that ZnO can act as a modifier (Anand et al., 2014; Balasubramanian et al., 2015), leading to the decrease in surface area and pore size of glass. Moreover, when present in higher amount, ZnO can also act as an intermediate oxide, thus creating a more stable glass structure by forming covalent links between adjacent SiO_4_ tetrahedra (El-Kady and Ali, 2012; Balasubramanian et al., 2015). Regarding ZnO role in glass bioactivity and degradation, Kamitakahara et al. (2006) reported that the introduction of ZnO (replacing CaO) in a bioactive apatite–wollastonite containing glass-ceramic decreases the glass-ceramic bioactivity. They reported that the glass-ceramic chemical durability was improved by adding ZnO because ZnO is an amphoteric oxide and shows very low solubility in SBF. Similar results were obtained by Goel et al. (2013). The silicon release of the glass-ceramic decreased with increasing ZnO content, and as a consequence, the formation of silanol groups was suppressed. It has been observed that the presence of zinc causes a general reduction in ion leaching in the 45S5 BG composition (Lusvardi et al., 2009). This behavior was also confirmed by Aina et al. (2009); in this study, it is proven that high level of ZnO (20 mol%) caused a drastic reduction of 45S5 glass leaching activity and so HAp formation. A 5 mol% of ZnO addition causes only a delay in the HAp precipitation; however, in this case, Zn substituted all glass elements. Oudadesse et al. (2011) reported that only 0.1 wt% of Zn (replacing Ca and Na) inhibited the glass dissolution, limiting the formation of silica gel layer and generating a delay in the HAp formation.

The present study confirms that Zn introduction retards the glass degradation and the crystal nucleation of HAp, even if for glass powders the silica gel formation and its enrichment in Ca and P were observed after few days of SBF immersion.

Data regarding Sr introduction in glasses has shown that its addition does not result in any structural alteration of the glass network (Fredholm et al., 2010), since its role is similar to the calcium one. Moreover, in general, this element does not modify or even enhances the bioactivity of the pristine glass (Lao et al., 2008, 2009). Only few studies reported a negative influence of Sr in HAp nucleation. Hoppe et al. (2014) reported that Sr-containing BG (type 1393) nanoparticles showed a delay in the bioactivity mechanism by increasing the Sr content, and Goel et al. (2011) showed that increasing the Sr2^+^/Ca^2+^ ratio in the glasses does not affect their structure significantly, but the apatite-forming ability is decreased considerably. In the present study, Sr introduction entails a slight delay in the bioactivity kinetics, but XRD (Figure 1B) and FESEM-EDS analyses (Figures 3B and 4B) demonstrated that after 3 days of SBF immersion, HAp crystals were present on the surface of the glass powders. It must be underlined that in the literature there are some controversies about the effect of Sr/Ca substitution on the dissolution mechanism of the glasses. These controversies seem to be due to the mixed use of weight instead of mole percent in glasses composition design (O’Donnell and Hill, 2010; Du and Xiang, 2012). In fact, if weight percentage is used, the higher molecular weight of SrO than CaO can lead to an actual increase of SiO_2_ content in mole percent, which involves an increase of network connectivity and a decrease of dissolution rate. Instead, if Sr is substituted in mole percent, the network structure of the glass does not significantly change and a higher dissolution rate is usually observed (Neel et al., 2009; O’Donnell and Hill, 2010)

Finally, the substitution of both Zn and Sr leads to an intermediate behavior; the bioactivity process of 45S5-ZnSr glass is obviously delayed; however, after 1 month in SBF solution, the performed XRD and FESEM-EDS analyses (Figures 1C and 4C) demonstrated the formation of a crystalline HAp layer on glass powders surface. Differently, Kapoor et al. evidenced HAp formation in few hours on Sr- and Zn-doped alkali-free glass, however with very different composition respect to the ones of the glasses investigated here (Kapoor et al., 2014).

The bioactivity test of composite coatings produced by EPD leads to the same observations: Zn introduction significantly modified the glass reactivity toward SBF solution while Sr introduction allows maintaining the bioactivity of the coatings.

By tuning the EPD parameters, it was possible to obtain composite coatings using all doped glasses with glass particles or clusters well embedded in the chitosan matrix; however, the glass composition seems to have an effect not only on the coating bioactivity but also on their deposition and adhesion to the metallic substrate; in fact, Zn-containing coatings showed qualitative low adhesion with respect to Sr-containing ones (Figure 7).

Moreover, some differences were observed between AC and DC deposition; usually, the AC-EPD technique allowed deposition of composite coating without the formation of cracks and bubbles while some bubbles were observed using DC-EPD, specially for Zn-containing coatings. When water is used as solvent, the use of DC can originate water electrolysis, which leads to the formation of oxygen or hydrogen bubbles formation and their entrapment in the coating; in the AC-EPD technique, the voltage is periodically inverted, so the supplied energy is not sufficient to cause water hydrolysis, and consequent bubble formation, and the coatings appeared more homogeneous (Neirinck et al., 2009; Chávez-Valdez and Boccaccini, 2012; Chen et al., 2013). Moreover, AC-EPD allowed the deposition of a slightly greater amount of material than DC-EPD. As confirmed by Kollatha et al. (2013), AC-EPD is advantageous in depositing denser and less cracked coatings in comparison to DC-EPD. The bubble formation can also have some influence on the roughness of the coatings; nevertheless, the observed difference among roughness values can be due to the grater amount of material deposited on the substrates using AC-EPD.

## Conclusion

The introduction of Zn and Sr in the 45S5 BG composition leads to a different behavior in terms of bioactivity, both for glass powders and composite coatings. The presence of ZnO allowed silica gel formation and its enrichment in Ca and P after few days of SBF immersion but inhibited the formation and precipitation of HAp while SrO introduction allowed the formation of a crystalline HAp after 1–3 days of SBF treatment. All glasses were successfully used to synthesize composite coatings by means of EPD with chitosan as the biopolymer component by adjusting the process parameters; however, Zn-containing coatings showed low adhesion to the substrate in comparison with 45S5-Sr/chitosan coatings. The deposition using AC revealed better coating quality than DC-EPD technique, since it allowed the realization of compact coatings, reducing bubbles and cracks formation. In conclusion, Zn concentration in 45S5 BG should be tailored to not completely inhibit the bioactivity process but at the same time to allow its antibacterial and anti-inflammatory effect and its role in bone metabolism while Sr-containing coatings, due to the bioactivity and biological effect of Sr, are promising materials for orthopedic coatings. A deeper investigation should be carried out in future work to evaluate the Sr ability to stimulate bone formation through *in vitro* biological tests.

## Conflict of Interest Statement

The authors declare that the research was conducted in the absence of any commercial or financial relationships that could be construed as a potential conflict of interest.
